# Molecular Epidemiology of *mcr-1*-Positive *Escherichia coli* and *Klebsiella pneumoniae* Isolates: Results from Russian Sentinel Surveillance (2013–2018)

**DOI:** 10.3390/microorganisms10102034

**Published:** 2022-10-14

**Authors:** Valeria Shapovalova, Elvira Shaidullina, Ilya Azizov, Eugene Sheck, Alexey Martinovich, Marina Dyachkova, Alina Matsvay, Yulia Savochkina, Kamil Khafizov, Roman Kozlov, German Shipulin, Mikhail Edelstein

**Affiliations:** 1 Federal State Budgetary Institution, Centre for Strategic Planning and Management of Biomedical Health Risks, Federal Medical Biological Agency, 119121 Moscow, Russia; 2 Institute of Antimicrobial Chemotherapy, Smolensk State Medical University, 214019 Smolensk, Russia; 3 Central Research Institute of Epidemiology, 111123 Moscow, Russia

**Keywords:** antibiotic resistance, colistin, polymyxins, mobile colistin resistance, *mcr-1*, *Escherichia coli*, *Klebsiella pneumoniae*, plasmids, IncI2, IncX4

## Abstract

Background: The dissemination of mobile colistin resistance (*mcr*) genes is a serious healthcare threat because polymyxins represent “last-line” therapeutics for multi-drug-resistant Gram-negative pathogens. This study aimed to assess the prevalence of colistin resistance and *mcr* genes and characteristics of clinical *Escherichia coli* (*Eco*) and *Klebsiella pneumoniae* (*Kpn*) isolates and plasmids carrying these genes in Russia. Methods: A total of 4324 *Eco* and 4530 *Kpn* collected in the frame of sentinel surveillance in 2013–2018 were tested for susceptibility to colistin and other antibiotics using the broth microdilution method. *mcr* genes were screened by real-time PCR. Phylogeny, genomic features and plasmids of *mcr*-positive isolates were assessed using whole-genome sequencing and subsequent bioinformatic analysis. Results: Colistin resistance was detected in 2.24% *Eco* and 9.3% *Kpn*. Twenty-two (0.51%) *Eco* and two (0.04%) *Kpn* from distant sites carried *mcr-1.1*. Most *mcr-*positive isolates co-harbored ESBLs and other resistance determinants to various antibiotic classes. The *mcr*-positive *Eco* belonged to 16 MLST types, with ST359 being most common; *Kpn* belonged to ST307 and ST23. *mcr-1.1* was carried mainly in IncI2 (*n* = 18) and IncX4 (*n* = 5) plasmids highly similar to those identified previously in human, animal and environmental isolates. Conclusion: This study demonstrated a dissemination of “typical” *mcr*-bearing plasmids among diverse *Eco* and *Kpn* genotypes and across a wide geographic area in Russia. Given the frequent association of *mcr* with other resistance determinants and potential clinical impact, the continual surveillance of this threat is warranted.

## 1. Introduction

Polymyxins (including colistin, or polymyxin E and polymyxin B), the cationic peptide antibiotics, were discovered more than 70 years ago and become clinically available in 1960s; however, their therapeutic use was long since abandoned because of nephrotoxicity and availability of alternative effective and better-tolerated antimicrobial agents [1]. In the last two decades, however, the emergence and global rise in resistance to all classes of antibiotics, including extended-spectrum cephalosporins and carbapenems, in Gram-negative bacteria have led to the reintroduction of polymyxins as “last-resort” drugs against extremely multi-resistant pathogens.

Polymyxins exert their antimicrobial action via initial interaction with negatively charged lipid A components of the lipopolysaccharide (LPS) in the outer membrane of Gram-negative bacteria. Until 2015, all known colistin resistance mechanisms were chromosomally mediated and usually related to mutations in several genes included in the regulation (*pmrAB*, *phoPQ*, and *mgrB*), biosynthesis (*pmrHFIJKLM*) and structural modification (*pmrC* and *pmrE*) of lipid A [2,3]. However, the discovery by Liu et al. [4] of the plasmid-borne mobile colistin resistance (*mcr-1*) gene for lipid A phosphoethanolamine transferase revealed a mechanism for horizontal spread of polymyxin resistance. Since then, *mcr-1* was detected in various species of Enterobacterales from different sources (humans, animals and the environment) and different countries across Asia, Europe, Africa, the Americas and Oceania. Notably, a retrospective screening of the collection of poultry *E. coli* isolates by the same research group that discovered *mcr-1* revealed its presence in isolates collected back in the 1980s when colistin first started to be used in food-producing animals in China [5]. In addition, several new variants of *mcr* genes sharing from 31% to 83% amino acid sequence identity to *mcr-1* have been identified in different Gram-negative bacteria, with *mcr-1* being the most frequently detected [6,7,8]. These genes have been associated with plasmids of diverse incompatibility types, among which, IncI2, IncHI2 and IncX4 were the most abundant [9].

Whole-genome sequencing (WGS) has become an essential tool for surveillance and molecular epidemiology of antimicrobial resistance (AMR). It provides precise spatial and temporal delineations of the spread of pathogens and mobile resistance genes. Coupled with epidemiological and phenotypic antimicrobial susceptibility investigations, it delivers ultimate resolution for tracing AMR [10]. Numerous studies have used WGS and bioinformatics analysis to characterize *mcr*-bearing plasmids and strains. Short-read sequencing (e.g., with Illumina platform) has been commonly applied to assess phylogenetic relationship of isolates based on analysis of core genome and to determine the presence of plasmids, virulence and AMR genes, while long-read sequencing (e.g., with the Oxford Nanopore platform) has been used to effectively resolve complex plasmid structures and genomic context of AMR genes. A hybrid assembly with short and long reads has been used to provide complete and accurate bacterial genomes [11].

Several research groups have previously reported on the sporadic occurrence of *mcr-1* in Russia and described the corresponding *mcr*-bearing plasmids [12,13,14,15]. In this study, we aimed to systematically assess the prevalence of colistin resistance and of *mcr* genes in clinical *Escherichia coli* and *Klebsiella pneumoniae* isolates from Russian sentinel surveillance and to determine phenotypic and genomic characteristics of *mcr*-carrying isolates and plasmids.

## 2. Materials and Methods

### 2.1. Bacterial Isolates

All studied isolates and accompanying metadata were collected as part of the national sentinel surveillance program of antimicrobial resistance in bacterial pathogens isolated from hospitalized patients [16]. Between 1 January 2013 and 31 December 2018, participating laboratories from 36 cities across Russia contributed a total of 4324 *E. coli* and 4530 K. pneumoniae clinical isolates. The isolates were recovered from representative clinical specimens (blood, tissue biopsies, cerebrospinal fluid, bronchoalveolar lavage, sputum, urine, etc.) of patients with clinical symptoms of infections; isolates from surface swab specimens, screening, and environmental samples were spared. Only one isolate of each species per patient/case of infection was allowed.

### 2.2. Species Identification, Antimicrobial Susceptibility Testing and PCR Screening of mcr Genes

In the central surveillance laboratory, the species identity of the isolates was confirmed by MALDI-TOF mass spectrometry with the Microflex LT-MALDI Biotyper System (Bruker Daltonics, Bremen, Germany).

The susceptibility to a range of antibiotics, including ampicillin, amoxicillin-clavulanic acid, piperacillin-tazobactam, ceftazidime, ceftazidime-avibactam, cefepime, aztreonam, cefotaxime, ertapenem, imipenem, meropenem, gentamicin, amikacin, ciprofloxacin, doxycycline, tigecycline, chloramphenicol, trimethoprim-sulfamethoxazole, fosfomycin, and colistin, was determined by a reference broth microdilution method according to ISO 20776-2:2021 [17] and European committee on antimicrobial susceptibility testing (EUCAST) [18] methodology. Susceptibility testing results were interpreted according to EUCAST v.12.0 Clinical breakpoints [19].

All isolates with colistin minimum inhibitory concentration (MIC) >0.5 mg/L (1105 *E. coli* and 1483 *K. pneumoniae*) were screened for the presence of *mcr* genes by means of real-time PCR as described elsewhere [20].

### 2.3. Whole-Genome Sequencing

Twenty-four *mcr*-positive isolates were characterized by WGS. Genomic DNA was extracted using DNeasy Blood & Tissue Kit and QIAcube Connect Device (QIAGEN, Hilden, Germany). For short-read sequencing, library preparation was performed using Illumina DNA prep kit (Illumina, San Diego, CA, USA) according to manufacturer instructions. Capillary electrophoresis using the Agilent 2100 Bioanalyzer system and Agilent High Sensitivity DNA Kit (Agilent, Santa Clara, CA, USA) was used for estimation of library quality. Concentration of the libraries was measured using the Qubit 4.0 fluorimeter with Qubit dsDNA HS Assay Kit (Thermo Fisher Scientific, Waltham, MA, USA). Sequencing was performed on the Illumina NextSeq 550 System with the NextSeq 500/550 Mid Output Kit v2.5 (300 Cycles) and on the Illumina MiSeq System with MiSeq Reagent Kit v2 (500-cycles) (Illumina, USA). For long-read sequencing, library preparation was performed with NEBNext Companion Module (New England Biolabs, Ipswich, MA, USA) and Ligation Sequencing Kit (Oxford Nanopore Technologies (ONT), Oxford, UK) in accordance with manufacturer’s protocol. Samples were multiplexed using Native Barcoding Expansion 1–12 (ONT, UK). Library concentration was measured with Qubit dsDNA HS Assay Kit and Qubit 4 Fluorometer (Thermo Fisher Scientific, USA). Sequencing was performed on the MinION platform and R9.4.1 Flow Cells (ONT, UK).

### 2.4. Bioinformatics Analysis

Quality assessment of raw reads was performed using a FASTQC v.0.11.9 tool [21]. The bioinformatics pipeline TORMES v.1.2.1 [22] was used for the reads quality filtering with Trimmomatic v.0.39 [23] and Prinseq v.0.20.4 [24], de novo genome assembly with Spades v.3.14.1 [25], quality assessment of assemblies with Quast v.5.0.2 [26], and taxonomic identification by k-mer matching with Kraken2 v2.1.1 [27] and 16S rRNA gene analysis with RDP Classifier v.2.10.2 [28]. All steps were performed with default parameters, except for Spades assembly, which was run with the flag --cov-cutoff set to ‘auto’ and the additional flag --careful. The minimum contig size threshold was set to 500 bp in length. Contamination removal was performed based on determining the coverage cutoffs for each assembly after visual inspection of Coverage-vs.-Length (CVL) plots as described by Douglass et al. [29]. PlasmidSPAdes [30] was used for de novo reconstruction of plasmids.

Base-calling and demultiplexing were conducted using Guppy v5.0.16 with the dna_r9.4.1_450bps_sup model. Quality control of sequencing was assessed with MinIONQC v1.4.2 [31]. Hybrid assemblies were prepared by using the Trycycler pipeline v0.5.1 [32], which included quality-control filtering of short reads by fastp v0.20.1 [33] with default parameters and filtering of long reads by Filtlong v0.2.0 [34] with a minimum read length of 1 kbp and a kept-base percentage of 95%. Long reads were randomly subsampled into 12 read sets, which were assembled using three different assemblers: Flye v2.9 [35], Miniasm 0.3/Minipolish v0.1.3 [36], and Raven v1.6.1 [37]. The contigs from different assemblies were then clustered by Trycycler to produce a consensus contig for each cluster. The consensus contigs were polished with Medaka v.1.4.4 [38]. Short-read polishing was performed by Polypolish tool v0.5.0 [39] and POLCA script [40]. Quast tool [26] was used to obtain the metrics of the final hybrid assemblies. The genome completeness of each assembly was assessed by performing a benchmarking universal single-copy ortholog (BUSCO) analysis using BUSCO 4.0.6 [41]. The level of genome completeness was expressed as BUSCO scores, including complete, fragmented, and missing BUSCOs that indicate the fractions of high-identity full-length genes, partially present genes, and absent genes, respectively. Gene prediction was performed for complete genome sequences with the PROKKA v1.14.5 [42]. Phylogroup, sequence type, serotype, and FimH type of *E. coli* were identified using ClermonTyping [43], MLST v2.1 [44] with PubMLST typing database [45], SerotypeFinder v.2.0 [46], and FimTyper v.1.1 [47]. Kleborate v2.1.0 [48] was used for K and O antigen typing of *K. pneumonia* genomes. Acquired antimicrobial resistance genes and known resistance-associated point mutations were annotated using AMRFinderPlus tool v. 3.10.5 [49] with BLASTP, BLASTX and HMMER algorithms and ABRicate tool v1.0.1 [50] with Resfinder database [51]. Searches for virulence factor genes and plasmid replicons were performed using the ABRicate with EcOH database [52], VFDB [53], PlasmidFinder [54] and ISFinder databases [55]. The hybrid assemblies were additionally checked for the presence of multiple copies of AMR genes using BLASTN 2.4.0+ [56]. The core genomes were defined by Prokka and Roary [57] and the resulting core-genome alignment file from Roary was used as input into IQTREE v.2.2.0.3 [58] to construct a phylogenetic tree based on the best fit model (GTR+F+I+I+R7) identified by ModelFinder [59]. Bootstrapping was performed with 100 replicates and with parameter-b. The branches had bootstrap values above 98 for branch support. Core-genome single-nucleotide polymorphisms (SNPs) were extracted from the core-genome alignment using the SNP-sites v.2.5.1 [60]. iTol v5 [61] was used for visualizing the trees. Plasmids were reconstructed by Spades or plasmidSpades preferably from hybrid assemblies or from the longest contigs of two independent short-read assemblies. MOB-suite tool [62] was also used to assist in plasmid reconstruction in cases where *mcr* and plasmid replicon genes were on different contigs Alignment and circular comparisons of plasmids was performed with BRIG v0.95 [63]. The National Center for Biotechnology Information (NCBI) Basic Local Alignment Search Tool (BLAST) was used to identify published plasmid sequences most similar to those reported herein.

### 2.5. Data Availability

All Illumina and MinION sequences were deposited in the European Nucleotide Archive under the project accession number PRJEB53051.

## 3. Results

**Figure 1:**
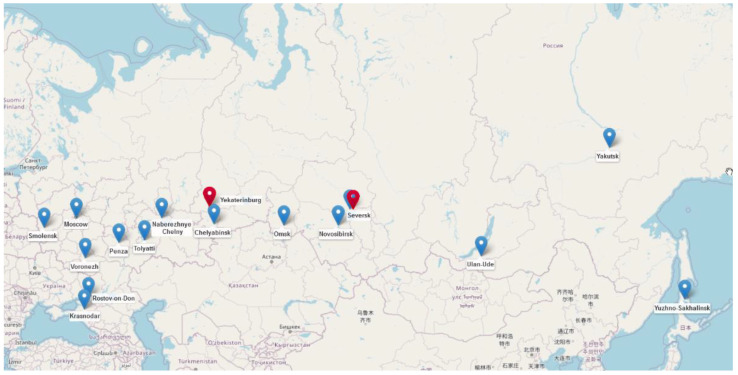
Map showing the geographic origins of *mcr*-positive *E. coli* (blue markers) and *K. pneumoniae* (red markers) isolates from this study.

A total of 4324 *E. coli* and 4530 *K. pneumoniae* consecutive non-duplicate clinical isolates collected in 36 cities across Russia between 1 January 2013 and 31 December 2018 were assessed for susceptibility to colistin and for the presence of *mcr* genes. Phenotypic colistin resistance was detected in 97 (2.24%) *E. coli* and 420 (9.3%) *K. pneumoniae* isolates, while the presence of *mcr* genes was detected in 22 (0.51%) *E. coli* and 2 (0.04%) *K. pneumoniae* isolates. The geographic origins of *mcr*-positive isolates are shown in Figure 1. These isolates represented community-onset (*n* = 8) and hospital-onset (*n* = 16) infections of the urinary tract (*n* = 8), abdominal cavity (*n* = 7), lower respiratory tract (*n* = 5), skin and soft tissue (*n* = 3) and bloodstream (*n* = 1). Detailed clinical information on these isolates is presented in Supplementary Table S1.

The colistin MICs of *mcr*-positive isolates ranged between 4 and 16 mg/L. In addition to colistin, *E. coli* isolates were resistant to multiple antibiotics, including (in order of decreasing occurrence) ampicillin (*n* = 21), ciprofloxacin (*n* = 21), cefotaxime (*n* = 17), aztreonam (*n* = 16), amoxicillin-clavulanic acid (*n* = 16), cefepime (*n* = 14), trimethoprim-sulfamethoxazole (*n* = 14), chloramphenicol (*n* = 12), ceftazidime (*n* = 10), gentamicin (*n* = 10), tigecycline (*n* = 6), fosfomycin (*n* = 4), piperacillin-tazobactam (*n* = 3), and amikacin (*n* = 1), but were all susceptible to carbapenems and ceftazidime-avibactam. The two *mcr*-positive *K. pneumoniae* isolates were resistant to ampicillin, amoxicillin-clavulanic acid, piperacillin-tazobactam, cefotaxime, ceftazidime, cefepime, aztreonam, ciprofloxacin, gentamicin and chloramphenicol, but were susceptible to imipenem, meropenem, ceftazidime-avibactam, amikacin and fosfomycin at EUCAST clinical breakpoints. One *K. pneumoniae* isolate was additionally resistant to ertapenem and the other one to trimethoprim-sulfamethoxazole (Supplementary Table S1).

Illumina short-read whole-genome sequencing was performed on all 24 *mcr*-positive isolates. The summary of Illumina sequencing reads and assembly statistics are provided in Supplementary Table S2. The number of contigs in genome assemblies ranged from 70 to 269, while the N50 of contigs ranged from 43 kb to 349 kb. Five *E. coli* isolates (ec_75812, ec_76498, ec_92380, ec_102833, ec_103195) and one *K. pneumoniae* (kp_87441) were selected for complementary long-read sequencing using Oxford Nanopore Technologies (ONT) MinION platform to generate complete chromosome and plasmid assemblies (Supplementary Table S3). The total number of contigs in the resulting hybrid assemblies combining ONT and Illumina reads ranged from 5 to 6 per assembly. The largest contig of *K. pneumoniae* isolate was 5.376 Mb, and the largest contig of each *E. coli* isolate was >4.7 Mb, indicating that chromosomal DNA of each isolate was assembled into a single contig (Supplementary Tables S3 and S4). In addition, a quality check was performed using BUSCO software to ensure >99% completeness (Supplementary Table S3).

**Figure 2:**
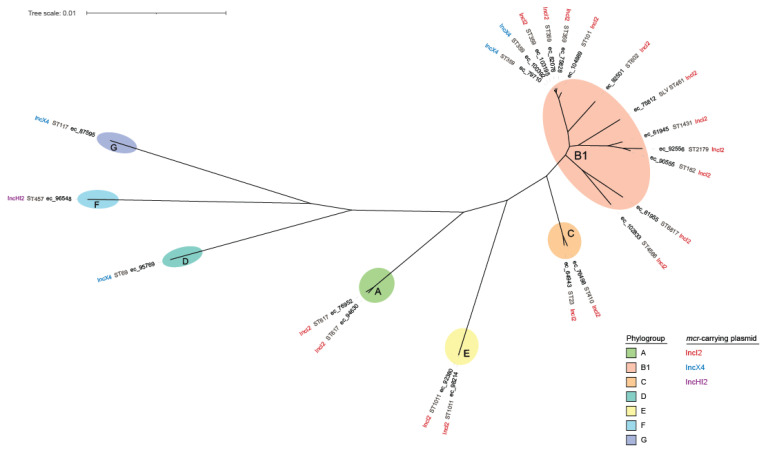
Core-genome SNP tree of 22 mcr-positive *E. coli* isolates. Sequence types (STs) and mcr-plasmid types are indicated next to the isolate names. Different *E. coli* phylogroups are shown by colored ellipses.

For all sequenced *E. coli* isolates, a core genome alignment of 2,976,673 bases was produced from 3066 (≥99%) core genes, yielding a final alignment of 150,635 single-nucleotide polymorphism (SNP) sites. In silico analysis assigned these isolates to seven different phylogroups (A, B1, C, D, F, G, and E) and 16 multilocus sequence types (STs) (Table 1, Figure 2). Notably, three STs included more than one epidemiologically unrelated isolates collected in geographically distinct regions: ST359 with five *E. coli* isolates (ec_79710, ec_100392, ec_103195, ec_75828, and ec_82078), the first three of which had the same serotype O36:H31 and carried *fimH35* allele; ST1011 with two isolates (ec_92380 and ec_98214), which belonged to serotype H28:O188 and *fimH31* type; and ST617 with two isolates (ec_76952 and ec_94630), which belonged to different serotypes and *fimH* types. One isolate (ec_75812) was found to belong to a new ST that differed from the most closely related ST461 at a single (*adk*) locus.

The two *K. pneumoniae* isolates (kp_87441 and kp_95329) belonged to ST307 and ST23 and carried the capsular biosynthesis loci (KL) KL102 and KL57, respectively (Table 2). Notably, the latter isolate carried typical for ST23 genotype hypervirulence marker genes, including aerobactin biosynthesis and receptor genes (*iucABCD-iutA*) and regulators of mucoid phenotype (*rmpA* and *rmpA2*).

Analysis of antibiotic resistance genes (ARGs) confirmed the presence of *mcr-1.1* variant gene in all sequenced *E. coli* and *K. pneumoniae* genomes. Consistent with phenotypic antibiotic susceptibility patterns, multiple ARGs to various antibiotic classes were found. (Table 1 and Table 2).

All but one *E. coli* genomes contained acquired β-lactamase genes, including class A and D penicillinase genes: *bla*_TEM-1_ (*n* = 13) and *bla*_OXA-1_ (*n* = 1) and, most notably, the genes for various CTX-M-type extended-spectrum β-lactamases (ESBLs): *bla*_CTX–M–15_ (*n* = 5), *bla*_CTX–M–1_ (*n* = 4), *bla*_CTX–M–55_ (*n* = 3), *bla*_CTX–M–65_ (*n* = 2), *bla*_CTX–M–27_ (*n* = 1), *bla*_CTX–M–169_ (*n* = 1), and genes for acquired AmpC cephalosporinases: *bla*_CMY-2_ (*n* = 2). All *E. coli* isolates also carried one or several ARGs to multiple classes of non-β-lactam antibiotics, aminoglycosides (*n* = 19) (*aadA1, aadA2, aadA5, aadA16, aac(3)-IId, aac(3)-IVa, aac(3)-VIa,* *aac(6′)-Ib-cr, aph(3′)-Ia, aph(3′)-Ib, aph(3′)-Id, aph(3′)-IIa, armA*), quinolones (*n* = 7) (*aac(6′)-Ib-cr, qnrB6, qnrB19, qnrS1*), sulphonamides (*n* = 18) (*sul1*, *sul2*, *sul3*), trimethoprim (*n* = 14) (*dfrA12*, *dfrA14, dfrA17, dfrA27*), macrolides (*n* = 11) (*mph(A), mph(E), msr(E)*), phenicols (*n* = 11) (*catA1*, *catB3, cmlA1, floR*), tetracyclines (*n* = 16) (*tetA, tetB, tetM*), fosfomycin (*n* = 2) (*fosA3*, *fosA7.5*), and rifampicin (*n* = 3) (*arr-3*), as well as known mutations in the quinolone resistance-determining regions (QRDRs) of *gyrA* (*n* = 21) (S83L, D87N, D87Y), *parC* (*n* = 21*)* (S80I, S80R, E84G), and *parE* gene (*n* = 8) (S458A), and mutations in the fosfomycin resistance-determining region of *uhpT* (*n* = 3) (E350Q) and *cyaA* gene (*n* = 3) (S352T).

The genomes of two *K. pneumoniae* isolates contained, in addition to chromosomal species-specific penicillinase (*bla*_SHV-1_ or *bla*_SHV-28_) and glutathione_transferase (*fosA*) genes, the genes for acquired penicillinases (*bla_OXA-_*_1_ and *bla*_LAP*-2*_ or *bla_TEM-1_*), ESBLs (*bla*_CTX-M-15_ or *bla*_CTX-M-55_), as well as various aminoglycoside (*aadA1, aadA2, aac(3)-IIe, aac(6′)-Ib-cr, aph(3″)-Ib, aph(6)-Id*), tetracycline (*tetA*), sulfonamide (*sul2, sul3*), trimethoprim (*dfrA14*), phenicol (*catB3, cmlA1*), quinolone (*aac(6′)-Ib-cr, oqxA, oqxB, qnrB1, qnrS1*) ARGs and point mutations in *gyrA* (S83I) and *parC* (S80I) QRDRs.

Interestingly, using ONT long-read sequencing revealed the presence of multiple copies of certain ARGs in the individual sequenced genomes. Thus, a region of three tandem repeats containing *aac(6′)-Ib-cr*, *bla_OXA-_*_1_, *catB3::IS26*, *tetR/tetA* and ΔTn*As1* was found in IncFIB plasmid of *K. pneumoniae* isolate kp_87441, which was supported by 20 individual long sequencing reads spanning the entire repeat region (Supplementary Figure S1, Supplementary Table S5). Two copies of *bla*_CTX–M–15_ were found in the IncM2 plasmid of *E. coli* isolate ec_92380, with six individual long reads spanning the ARG region (Supplementary Figure S2, Supplementary Table S6); and two copies of *bla*_TEM–1_, one of which was carried on IncFIB(AP001918)/IncQ1/IncFIC(FII) plasmid and the other on the chromosome, were found in another *E. coli* isolate, ec_76498.

Analysis *mcr-1.1* gene context revealed its association with plasmids of three replicon types: (i) IncI2 in 14 *E. coli* and 1 *K. pneumoniae*, (ii) IncX4 in 3 *E. coli* and 1 *K. pneumoniae*, and (iii) IncHI2 in a single *E. coli* isolate (Table 1 and Table 2). *Mcr-1.1* gene was detected on the same contig with IncI2 plasmid marker genes in 17 isolates and with IncX4 plasmid marker genes in 5 isolates. The *mcr*-bearing plasmids of the remaining two isolates were partially reconstructed using MOB-suite and were assigned to replicon types IncI2 and IncHI2.

**Figure 3:**
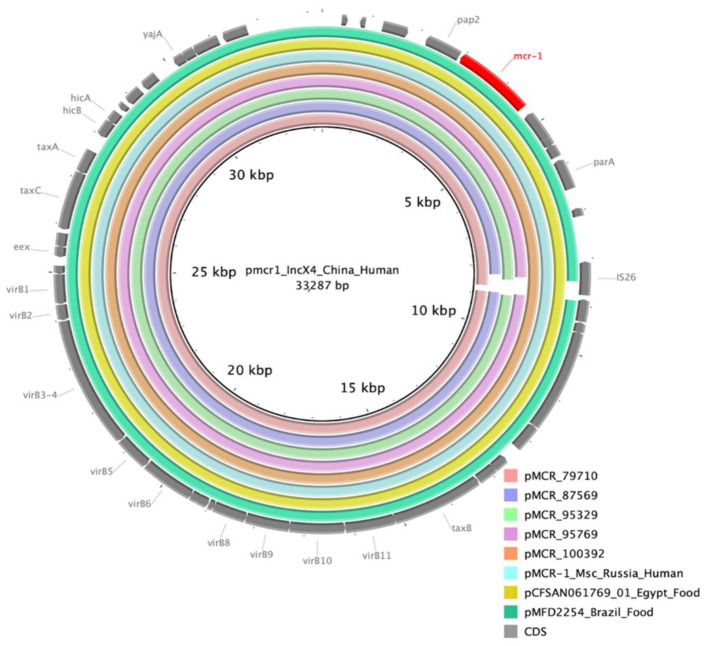
Sequence alignment of five IncX4 *mcr*-bearing plasmids from this study and *references* from GenBank. The sequence of IncX4 plasmid pmcr1 (GenBank acc. no. KU761327) was used as a reference (inner circle). The concentric circles show similar plasmids from this study and GenBank references and are colored according to legend. Gaps in the circles correspond to plasmid regions which are missing compared to the reference. The key plasmid genes and mcr-1.1 are shown in the outer circle with gray and red arrows.

The size of the five IncX4 plasmids varied between 32,744 and 33,437 bp. A BLAST search against the NCBI nr/nt database showed high-nucleotide sequence identity (99% to 100%) and >99% coverage between these plasmids and the known *mcr*-bearing IncX4 plasmids reported from diverse epidemiological sources and geographic locations across the globe, such as pCFSAN061769_01 (GenBank acc. no. CP042970.1) of *E. coli* isolated from raw milk cheese in Egypt, pMCR-1_Msc (GenBank acc. no. NZ_MK172815.1) of *E. coli* isolated from human respiratory specimen in Moscow, Russia, pMFDS2254 (GenBank acc. no. MK869758.1) of *E. coli* isolated from chicken meat in Brazil, and pCFSA231 (GenBank acc. no. CP033349.2) of *Salmonella enterica* isolated from food sample in China. In addition to similar size and common backbone structure, all the above IncX4 plasmids showed the absence of IS*Apl1* element up- and downstream of *mcr-1.1* and differed mainly in the presence or absence of IS*26* element (Figure 3).

**Figure 4:**
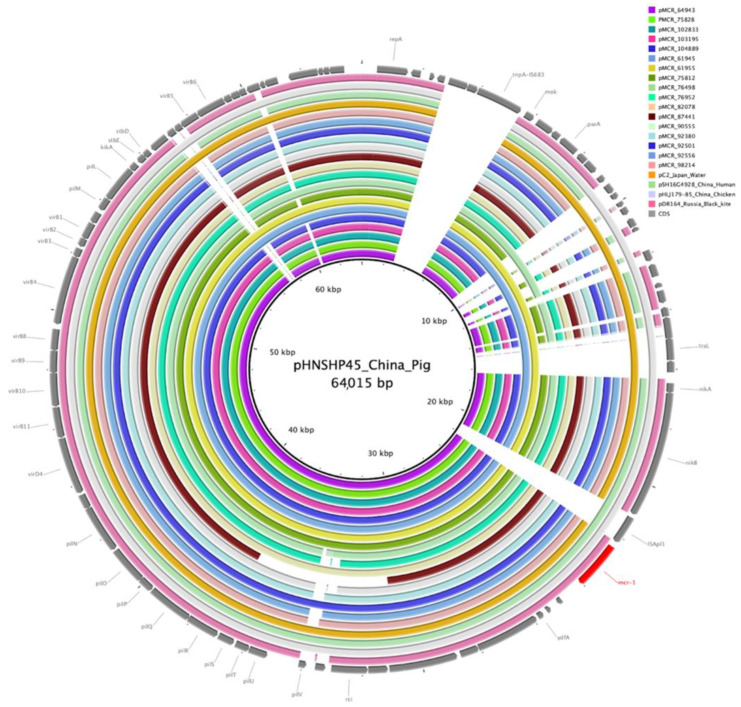
Sequence alignment of seventeen IncI2 *mcr*-bearing plasmids from this study and *references* from GenBank. The sequence of IncI2 plasmid pHNSHP45 (GenBank acc no. KP347127) was used as a reference (inner circle). The concentric circles show similar plasmids from this study and GenBank references and are colored according to legend. Gaps in the circles correspond to plasmid regions which are missing compared to the reference. The key plasmid genes and mcr-1.1 are shown in the outer circle with gray and red arrows.

The length of the contigs that contained *mcr-1.1* and IncI2 replicon genes ranged from 55,661 to 62,806 bp. These contigs contained typical IncI2 backbone elements, including the plasmid stability, replication, maintenance, and horizontal gene transfer regions. *mcr-1.1* gene was located immediately downstream of the *nikB* gene encoding plasmid relaxase without an upstream IS*Apl1*. The reported *mcr*-carrying IncI2 plasmids showed the highest (99% to 100%) sequence identity to the plasmids pC2 (GenBank acc. no. LC473131.1) of *E. coli* isolated from municipal wastewater in Japan, pDR164 (GenBank acc. no. MK542639.1) of *E.coli* isolated from wild bird in Russia, and several plasmids reported in China: pSh125-m2 (GenBank acc. no. KY363998.1) of *Shigella sonnei* and pSH16G4928 (GenBank acc. no. MH522426.1) of *Salmonella enterica* ser. Typhimurium isolated from human specimens, p5ZF15-2-1 (GenBank acc. no. CP079893.1) and pHLJ179-85 (GenBank acc. no. MN232214.1) of swine and chicken *Escherichia fergusonii* isolates (Figure 4).

## 4. Discussion

In this study, the prevalence of phenotypic colistin resistance and of the mobile colistin resistance (*mcr-*1) genes among clinical *E. coli* and *K. pneumoniae* isolates collected from hospitalized patients in Russia in 2013–2018 as part of sentinel surveillance was systematically assessed, and in-depth analysis of the genome sequences of *mcr*-bearing isolates and plasmids was performed. While there have been several reports of *mcr-1* positive isolates recovered from human and animal sources in Russia [12,13,14,15], to our knowledge, this study is the first to report the prevalence and genomic context of *mcr-1* in clinical isolates collected from geographically distant sites across Russia.

It should be noted that *mcr* has been observed even in isolates with colistin MICs below the clinical susceptibility breakpoint of ≤2 mg/L [63]. Therefore, in this study, PCR was used to screen for *mcr* in all isolates with colistin MIC of >0.5 mg/L to maximize the likelihood of detection. All *mcr*-positive isolates detected in this study, however, were resistant to colistin with MICs of 4 to16 mg/L. Phenotypic colistin resistance was detected in 2.24% of all *E. coli* and 9.3% of all *K. pneumoniae* isolates obtained in surveillance, but the prevalence of *mcr-1* was relatively low: 0.51% and 0.04% (22.68% and 0.48% among colistin-resistant isolates), respectively. Similar prevalence of colistin resistance (2.1% in all *Enterobacterales except Morganellaceae*) and *mcr* gene presence (42.19% among colistin-resistant *E. coli* and 0.21% among colistin-resistant *K. pneumoniae*) have been observed in the INFORM global surveillance program [64]. These data likely suggest that, in human clinical isolates, *mcr* is still a less common mechanism of resistance to polymyxins than chromosomal mutations. Though important, the analysis of mutational resistance mechanisms to polymyxins in *mcr*-negative isolates was out of scope of this study, which was specifically focused on characterization of *mcr*-positive isolates. Worryingly, a frequent association of colistin resistance with resistances to other classes of antibiotics among *mcr*-positive isolates, especially to oxyimino-cephalosporins due to the presence of ESBL (*bla*_CTX-M_) and acquired cephalosporinase (*bla*_CMY-2_) genes, and to fluoroquinolones due to the presence of plasmid-mediated *qnr* and *aac(6′)-Ib-cr* genes and mutations in chromosomal genes, was observed in this study, as well as in others [65]. One *E. coli* isolate was found to co-harbor a ribosomal methyltransferase (*armA*) gene conferring pan-aminoglycoside resistance. None of the isolates carried carbapenemase genes; however, one *K. pneumoniae* isolate exhibited resistance to ertapenem presumably mediated by overproduction of CTX-M-55 ESBL and porin alteration [66].

Despite relatively low prevalence, wide geographical dissemination of *mcr-1* was observed in Russia. All detected cases of infections due to *mcr*-positive isolates were epidemiologically unrelated and occurred in 17 hospitals in 16 geographically distant cities. The 22 *E. coli* isolates with *mcr-1* were assigned to seven different phylogroups and 16 STs. The major clonal group was composed of ST359 and its double-locus variant, ST101, included six isolates. Both ST359 and ST101 have been previously reported among *mcr*-positive isolates of human and animal origin in Asia, Europe, Americas and Oceania [67]. The two *K. pneumoniae* isolates with *mcr-1* were assigned to ST307 and ST23, which are both known as international ‘high-risk clones’ and have been commonly found in clinical settings in Russia [68,69,70,71,72,73]. To our knowledge, however, this is the first documented report of ST23 *K. pneumoniae* carrying both hypervirulence marker genes characteristic for this lineage and multiple acquired resistance genes, including *mcr-1.1* and *bla*_CTX-M-55_.

The *mcr-1* gene has been previously found in plasmids of more than 30 different replicon types, of which IncI2 and IncX4 have been the most common globally [74]. In this study, it was also detected mostly in IncI2 (*n* = 18) and IncX4 (*n* = 5) plasmids. Furthermore, significant structure and sequence similarity between the *mcr-1*-bearing IncI2 and IncX4 plasmids from this study and those reported earlier in isolates of different Enterobacterales species from different world regions and sources, including humans, livestock, wildlife animals, food and the environment, was observed. This once again confirms that the above plasmids are the main vehicle for *mcr-1* transmission. Of notice, stable genetic environment of *mcr-1* was found in all described plasmids and neither the presence of upstream IS*Apl1* element nor of the composite transposon Tn*6330* (IS*Apl1*-*mcr-1*-orf-IS*Apl1* structure), which has been proposed to be involved in initial mobilization and translocation of *mcr-1* between various plasmid backbones [75], was detected.

In summary, this report provides a systematic and comprehensive analysis of the prevalence and molecular epidemiology of *mcr-1*-positive *E. coli* and *K. pneumoniae* in Russia. Further to previous reports, it shows the wider geographic spread of *mcr-1*, despite low prevalence in clinical settings. Given the frequent association of *mcr-1* with other resistance determinants and its potential clinical impact, the continual surveillance of this threat is warranted.

## Figures and Tables

**Table 1:** Characteristics of *mcr-*positive *E. coli* isolates.

ID	Collection Date	Phylo-Group	ST	Serotype	*fimH* Type	Plasmid Type (Length of *mcr-1.1* Contig)	ARGs
ec_61945	1 January 2013	B1	ST1431	O8:H19	*fimH32*	IncI2 (60859 bp)	*mcr-1.1, blaCTX-M-1, aac(6′)-Ib-cr, arr-3, dfrA27, aadA16, qacEdelta1, sul1, sul2, tet(A)*
ec_61955	5 August 2013	B1	ST6817	O85:H23	*fimH31*	IncI2 (60860 bp)	*mcr-1.1, blaTEM-1, blaCTX-M-15, dfrA12, aadA2, cmlA1, aadA1, qacL, tet(A), tet(M), sul3, aph(3′)-Ia*
ec_64943	30 May 2014	C	ST23	O78:H4	*fimH35*	IncI2 (61030 bp)	*mcr-1.1, catA1, dfrA17, aadA5, tet(B), blaTEM-1, sul2, aph(3″)-Ib, aph(6)-Id, aph(4)-Ia, aac(3)-IVa, qnrB19*
ec_75812	1 June 2015	B1	new (SLV ST461)	O178:H28	*fimH31*	IncI2 (60903 bp)	*mcr-1.1, sul1, qacEdelta1, aac(3)-VIa, aadA1, qnrS1, blaCTX-M-15*
ec_75828	2 June 2015	B1	ST359	O23:H21	*fimH35*	IncI2 (61036 bp)	*mcr-1.1, aph(3′)-Ia, aph(6)-Id, aph(3″)-Ib, tet(B), blaTEM-1, sul2*
ec_76498	16 June 2015	C	ST410	O66:H45	*fimH24*	IncI2 (62806 bp)	*mcr-1.1, blaCTX-M-27, floR, aac(6′)-Ib-cr, arr-3, dfrA27, aadA16, qacEdelta1, sul1, mph(A), blaTEM-1, aac(3)-IId, bleO, dfrA17, catA1, tet(B), blaTEM-1, sul2, aph(3″)-Ib, aph(6)-Id, aph(3′)-Ia, blaCMY-2*
ec_76952	22 October 2015	A	ST617	O101:H10	*fimH29*	IncI2 (59391 bp)	*mcr-1.1, mph(A), catB3, blaOXA-1, aac(6′)-Ib-cr, aph(4)-Ia, aac(3)-IVa, aph(6)-Id, aph(3″)-Ib, tet(B), blaCTX-M-15, sul1, qacEdelta1, aadA5, dfrA17*
ec_79710	16 March 2016	B1	ST359	O36:H31	*fimH35*	IncX4 (33106 bp)	*mcr-1.1, blaCTX-M-1, tet(A), sul1, qacEdelta1, aac(3)-VIa, aadA1, aac(3)-IId, blaTEM-1, mph(A)*
ec_82078	9 June 2016	B1	ST359	O29:H31	*fimH35*	IncI2 (61024 bp)	*mcr-1.1, tet(A), dfrA12, mph(A), sul1, qacEdelta1, cmlA1, aadA1, qacL, sul3, aph(4)-Ia, aac(3)-IVa, aph(6)-Id, aph(3″)-Ib, aadA2*
ec_87595	15 March 2017	G	ST117	O45:H51	*fimH97*	IncX4 (32744 bp)	*mcr-1.1, blaCMY-2, mph(A), sul1, qacEdelta1, aadA2, dfrA12, tet(A), sul2, aph(3″)-Ib, aph(6)-Id*
ec_90555	7 March 2017	B1	ST162	O88:H21	*fimH32*	IncI2 (60006 bp)	*mcr-1.1, blaTEM-1, blaCTX-M-65, mph(A), sul1, qacEdelta1, aadA5, dfrA17, aac(3)-IId, floR, fosA3, aph(3″)-Ib, aph(6)-Id, tet(A), sul2*
ec_92380	17 July 2017	E	ST1011	O188:H28	*fimH31*	IncI2 (60934 bp)	*mcr-1.1, blaTEM-1, blaTEM-1, armA, msr(E), mph(E), blaCTX-M-15, blaCTX-M-15*
ec_92501	14 August 2017	B1	ST602	O-*:H21	*fimH86*	IncI2 (61163 bp)	*mcr-1.1, floR, blaTEM-1, aph(6)-Id, aph(3″)-Ib, dfrA17, aac(3)-IId, fosA7.5, sul2, catA1, blaCTX-M-169*
ec_92556	23 September 2017	B1	ST2179	O9/09a:H9	*fimH32*	IncI2 (59951 bp)	*mcr-1.1, blaTEM-1, blaCTX-M-65, tet(A), aph(6)-Id, aph(3″)-Ib, sul2, mph(A), floR*
ec_94630	10 October 2017	A	ST617	O101/O9:H9	*	IncI2 ** (23072 bp)	*mcr-1.1, blaCTX-M-55, blaTEM, aph(3′)-IIa, ble, dfrA14, aac(6′)-Ib-cr, arr-3, dfrA27, aadA16, qacEdelta1, sul1, qnrB6, qacEdelta1, sul1, aph(6)-Id, aph(3″)-Ib, sul2, floR*
ec_95769	28 November 2017	D	ST69	O15:H18	*fimH27*	IncX4 (32819 bp)	*mcr-1.1, blaCTX-M-15, qnrS1, blaTEM-1, aph(6)-Id, aph(3″)-Ib, sul2, tet(A), dfrA14*
ec_96548	1 November 2017	F	ST457	O11:H25	*fimH14*5	IncHI2 * (2325 bp)	*mcr-1.1, blaTEM-1, aadA2, tet(A), sul3, qacL, aadA1, cmlA1, floR, sul1, qacEdelta1, lnu(F), dfrA14, mph(A)*
ec_98214	22 March 2018	E	ST1011	O188:H28	*fimH3*1	IncI2 (60456 bp)	*mcr-1.1, blaTEM-1*
ec_100392	3 April 2018	B1	ST359	O36:H31	*fimH3*5	IncX4 (33437 bp)	*mcr-1.1, blaCTX-M-55*
ec_102833	28 April 2018	B1	ST4566	O163:H23	*fimH60*	IncI2 (61680 bp)	*mcr-1.1, tet(A), sul2, blaCTX-M-1*
ec_103195	27 September 2018	B1	ST359	O36:H31	*fimH35*	IncI2 (60820 bp)	*mcr-1.1, sul2, blaCTX-M-1, tet(A), mph(A), dfrA17, aac(3)-IId, blaTEM-1*
ec_104889	31 October 2018	B1	ST101	O115:H31	*fimH86*	IncI2 (61030 bp)	*mcr-1.1, aph(3″)-Ib, aph(6)-Id, blaCTX-M-55, catA1, dfrA14, mph(A), tet(B)*

* Not determined; ** Partial sequence.

**Table 2:** Characteristics of *mcr-*positive *K. pneumoniae* isolates.

ID	Collection Date	ST	K_Locus	O_Locus	Plasmid Type (Length of *mcr-1.1* Contig)	ARGs
kp_87441	9 February 2017	307	KL102	O2v2	IncI2 (55661 bp)	*mcr-1.1, oqxB19, oqxA, blaSHV-28, fosA, sul3, qacL, aadA1, cmlA1, aadA2, sul2, aph(3″)-Ib, aph(6)-Id, blaTEM-1, blaCTX-M-15, dfrA14, aac(3)-IIe, qnrB1, tet(A), catB3, blaOXA-1, aac(6′)-Ib-cr, tet(A), catB3, blaOXA-1, aac(6′)-Ib-cr, tet(A), catB3, blaOXA-1, aac(6′)-Ib-cr*
kp_95329	27 December 2017	23	KL57	O2v2	IncX4 (32850 bp)	*mcr-1.1, aac(3)-IIe, catB3, blaOXA-1, aac(6′)-Ib-cr, oqxA, oqxB, blaSHV-1, fosA, qnrS1, blaLAP-2, tet(A), sul2, blaCTX-M-55*

## Data Availability

All Illumina and MinION sequences and assemblies were deposited in the European Nucleotide Archive under the project accession number PRJEB53051.

## Supplementary Materials

The following supporting information can be downloaded at: https://www.mdpi.com/article/10.3390/microorganisms10102034/s1, Table S1. Clinical information and antibiotic susceptibilities of *mcr*-positive isolates. Table S2. Illumina sequencing read and assembly statistics. Table S3. MinION sequencing read statistics and hybrid assembly summary. Table S4. Features of hybrid assembly contigs. Table S5. Summary of MinION reads spanning the entire tandem repeat region in IncFIB(K) plasmid of *K. pneumoniae* kp_87441. Table S6. Summary of MinION reads spanning the region with two copies of *bla*_CTX-M-15_ gene in IncM2 plasmid of *E. coli* ec_92380. Figure S1. Structure of tandem repeat region in 175,835-bp, IncFIB(K) plasmid carried by of *K. pneumoniae* kp_87441. Figure S2. Structure of repeat region in IncM2 plasmid of *E. coli* ec_92380.
