# Supplementary material for: Molecular Epidemiology of mcr-1-Positive Escherichia coli and Klebsiella pneumoniae Isolates: Results from Russian Sentinel Surveillance (2013–2018)
Source: Microorganisms. 2022 Oct 14;10(10):2034. doi: 10.3390/microorganisms10102034 (PMC9607333; doi:10.3390/microorganisms10102034)
Supplement: Supplementary file 1 [file microorganisms-10-02034-s001.zip › Supplementary Tables_S2-S6_Figures_S1-S2.pdf]

Table S1. Clinical information and antibiotic susceptibilities of mcr-positive isolates. (see separate Excel file)

Table S2. Illumina sequencing read and assembly statistics.

Isolate ID	Assembly_acc. No.	Total reads	Total gigabases	Number contigs	Largest contig	Total length	N50
ec_61945	GCA_943733225	3785682	0.72	71	461404	5045330	208134
ec_61955	GCA_943733255	3194468	0.61	78	725547	5207996	300813
ec_64943	GCA_943737895	3848646	0.88	123	449094	5161458	162562
ec_75812	GCA_943733325	4246924	0.66	*	*	*	*
ec_75828	GCA_943733365	1933564	0.29	202	205693	5112990	53516
ec_76498	GCA_943733315	5202530	1.18	*	*	*	*
ec_76952	GCA_943733245	3828326	0.7	172	254543	5081776	103380
ec_79710	GCA_943733295	5392016	0.95	103	409089	5129795	138575
ec_82078	GCA_943733185	4323924	0.81	101	342010	5045558	150141
kp_87441	GCA_943733235	1983800	0.48	*	*	*	*
ec_87595	GCA_943733205	3960102	0.86	120	681689	5368066	216392
ec_90555	GCA_943733285	6007556	1.18	77	479865	5173826	246720
ec_92380	GCA_943733335	2702562	0.66	*	*	*	*
ec_92501	GCA_943733375	2056004	0.3	191	217513	5047406	72285
ec_92556	GCA_943733175	2349122	0.35	115	296868	5020356	135824
ec_94630	GCA_943733195	14409954	2.13	152	344384	4999490	89127
kp_95329	GCA_943733265	19974624	3	150	419737	5513988	91067
ec_95769	GCA_943733305	4531996	1.06	70	464828	5192104	349262
ec_96548	GCA_943737905	4572266	0.67	134	349967	5334064	113262
ec_98214	GCA_943733275	1087926	0.15	269	215530	5283010	43680
ec_100392	GCA_943733355	1986662	0.29	161	186342	4944825	78852
ec_102833	GCA_943733345	1414530	0.19	*	*	*	*
ec_103195	GCA_943733215	2379990	0.35	*	*	*	*
ec_104889	GCA_943737915	2125190	0.31	194	195851	5260242	70805

*see hybrid assembly statistics

Table S3. MinION sequencing read statistics and hybrid assembly summary.

ID	ec_75812	ec_76498	kp_87441	ec_92380	ec_102833	ec_103195
Total reads	79595	89995	520410	91293	348744	171282
Total gigabases	0.97	1.05	5.15	1.05	2.28	1.24
Mean length	12232.9	11715.2	9898.5	11551.7	6540.7	7263.7
Median length	8183	7169	6938	7777	4441	4907
# contigs	4	5	5	6	5	6
Largest contig	4771886	4807973	5376000	4978390	4785049	4787017
Total length	5087873	5283247	5839592	5395169	5052020	5186880
N50	4771886	4807973	5376000	4978390	4785049	4787017
BUSCO*	C:100.0%[S:99.2%,D:0.8%],F:0.0%,M:0.0%,n:366	C:100.0%[S:99.7%,D:0.3%],F:0.0%,M:0.0%,n:366	C:99.4%[S:98.9%,D:0.5%],F:0.0%,M:0.6%,n:366	C:100.0%[S:99.7%,D:0.3%],F:0.0%,M:0.0%,n:366	C:100.0%[S:99.7%,D:0.3%],F:0.0%,M:0.0%,n:366	C:100.0%[S:99.5%,D:0.5%],F:0.0%,M:0.0%,n:366

* C, Complete BUSCOs; S, Complete and single-copy BUSCOs; D, Complete and duplicated BUSCOs; F, Fragmented BUSCOs; M, Missing BUSCOs; n, Total BUSCO groups searched

Table S4. Features of hybrid assembly contigs.

Sample ID	Contig No.	Contig length, bp	Plasmid replicon marker genes	ARGs
kp_87441	1	5376000		*OqxB, OqxA, blaSHV-28, fosA6*
	2	216101	IncFIB (pNDM-Mar), IncHI1B (pNDM-MAR)	*sul3, ant(3'')-Ia, cmlA1, aadA2*
	3	175835	IncFIB(K)	*sul2, aph(3'')-Ib, aph(6)-Id, blaTEM-1B, blaCTX-M-15, dfrA14, aac(3)-IIa, qnrB1, tet(A), catB3::IS26, blaOXA-1, aac(6')-Ib-cr, tet(A), catB3::IS26, blaOXA-1, aac(6')-Ib-cr, tet(A), catB3::IS26, blaOXA-1, aac(6')-Ib-cr*
	4	55661	IncI2	*mcr-1.1*
	5	15995		
ec_92380	1	4978390		*blaTEM-1B, mdf(A), blaEC*
	2	170969	IncFIB, IncFIC(FII), IncFIA	*sitABCD*
	3	96645	IncB/O/K/Z	
	4	81317	IncM2	*blaTEM-1B, armA, msr(E), mph(E), blaCTX-M-15, blaCTX-M-15*
	5	60934	IncI2	*mcr-1.1*

	6	6914		
ec_76498	1	4807973		*mdf(A), blaEC*
	2	232781	IncHI1A, IncFIA(HI1), IncHI1B(R27)	*blaCTX-M-27, floR, aac(6')-Ib-cr, ARR-3, dfrA27, aadA16, qacE, sul1, mph(A), blaTEM-1B, aac(3)-IId*
	3	128890	IncFIB(AP001918), IncQ1, IncFIC(FII)	*bleO, dfrA17, catA1, tet(B), blaTEM-1B, sul2, aph(3'')-Ib, aph(6)-Id, aph(3')-Ia, sitABCD*
	4	62806	IncI2	*mcr-1.1*
	5	50797	IncB/O/K/Z	*blaCMY-2*
ec_75812	1	4771886		*mdf(A), blaEC*
	2	164981	IncFIB (AP001918)	*sul1, qacE, aac(3)-VIa, ant(3'')-Ia, sitABCD*
	3	90103	IncI1-I(Alpha)	*qnrS1, blaCTX-M-15*
	4	60903	IncI2	*mcr-1.1*
ec_102833	1	4785049		*mdf(A), blaEC*
	2	111693	IncFIB (AP001918), IncFII (pRSB107)	*sitABCD*
	3	88029	IncI1-I(Alpha)	*tet(A), sul2, blaCTX-M-1*
	4	61680	IncI2	*mcr-1.1*
	5	5569		
ec_103195	1	4787017		mdf(A), *blaEC*
	2	115504	IncI1-I(Alpha)	sul2, blaCTX-M-1
	3	114477	IncFIB (H89 - PhagePlasmid)	
	4	101575	IncFIB (AP001918)	tet(A), mph(A), dfrA17, aac(3)-IId, blaTEM-1B, sitABCD
	5	60820	IncI2	mcr-1.1
	6	7487		

Table S5. Summary of MinION reads spanning the entire tandem repeat region in IncFIB(K) plasmid of *K. pneumoniae* kp_87441.

Read id	Sequence length	Mean qscore
f2b5ff87-edfb-4467-a705-553f3ecce830	28050	13.278804
cf1cee3b-78d7-4696-93e2-75bde2d4cd14	38684	14.706444
7ee79274-79b0-4190-8459-642ebaa0731c	46501	12.657187
1cfa0eb7-b14e-45bb-9c15-7b9ebbb0f8cc	30312	12.069852
7920f6a2-b915-46cb-bc80-e706c5e3c7c7	42960	13.41891
21baa216-615f-4767-a97b-680af18e3028	38042	13.46641
d7ffbaa6-8d83-46ee-92bf-1e165779c6c1	46682	10.952122
b2de7e5e-9209-4cf3-a612-fe7cbbf403d6	28747	13.412342
cbcfa683-13cb-4c20-8b6a-ec569264c077	37650	13.379159
f849b679-78b6-46f1-a599-09f9f062bf57	24499	12.53458
25419807-36b5-4648-82bb-207c0ca81411	32670	16.785809
6f46aef7-b08f-4580-b09e-e49b58ab4bd2	26119	12.605803
c366f401-4c0a-4fde-aaf6-a932948f26ac	28168	10.227049
a34c5cdd-664b-4e02-8f32-77a010eac1c0	30727	13.766564
1e413d6e-34ef-4cc6-a74f-f1887c5e333d	32619	13.08557
abab6b07-5a48-42d2-8a6a-1ca8b86ee021	33406	15.00999
c18f7920-e534-49af-9380-f77c579b7aa9	34462	14.851232
c35c7293-96c6-4263-b742-a6e4ba8b679b	27983	14.957499
d4f0b683-c509-4d46-bc9d-d749edd841f8	51429	15.851901
3fa65401-7fd2-46cf-b2c6-0640d6fc0c42	48113	14.167575

Table S6. Summary of MinION reads spanning the region with two copies of *bla*$_{CTX-M-15}$ gene in IncM2 plasmid of *E. coli* ec_92380.

Read id	Sequence length	Mean qscore
64914c1f-eafe-4ff4-a128-39e9ef2aaa4d	35062	14.90551
b1a37b39-695b-407a-b482-aa90bcb98940	40154	13.464262
0e1be333-1707-4688-aa79-5f48db46a002	52727	12.337336
50315b36-e88d-4586-acef-906c59fa6f65	18745	13.603369
0e93ebe0-17bc-4411-8f98-e6c320b9030c	29886	13.814285
5688ca87-5b0b-494f-a212-d7fb0ca9525a	62808	14.253023

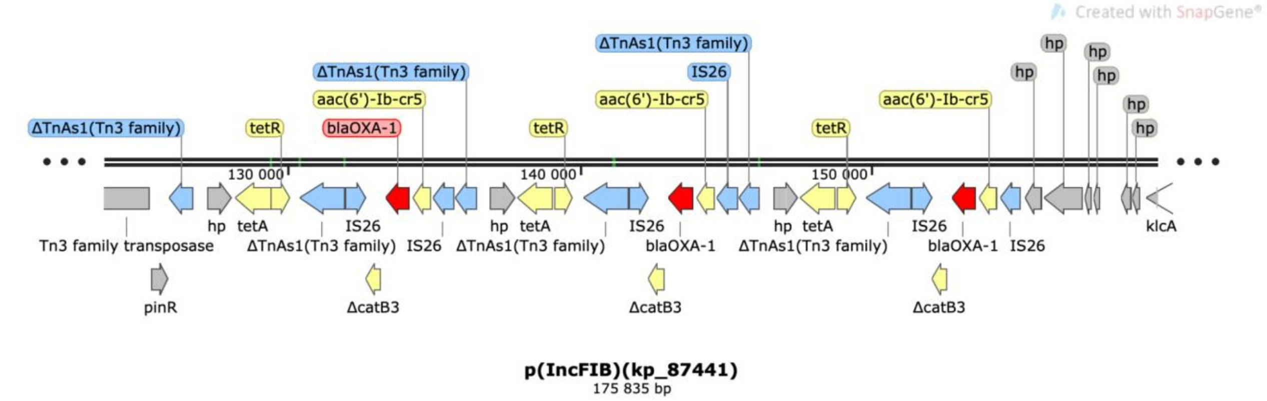

Figure S1. Structure of tandem repeat region in 175,835 bp, IncFIB(K) plasmid carried by of *K. pneumoniae* kp_87441.

Open reading frames (ORFs) and their transcriptional orientation are shown by arrows with respective annotations. ORFs are colored as follows: *bla*$_{OXA-1}$ (red), other ARGs (light yellow), IS/Tn elements (light blue), other genes (grey). Delta (Δ) indicates partial deletion.

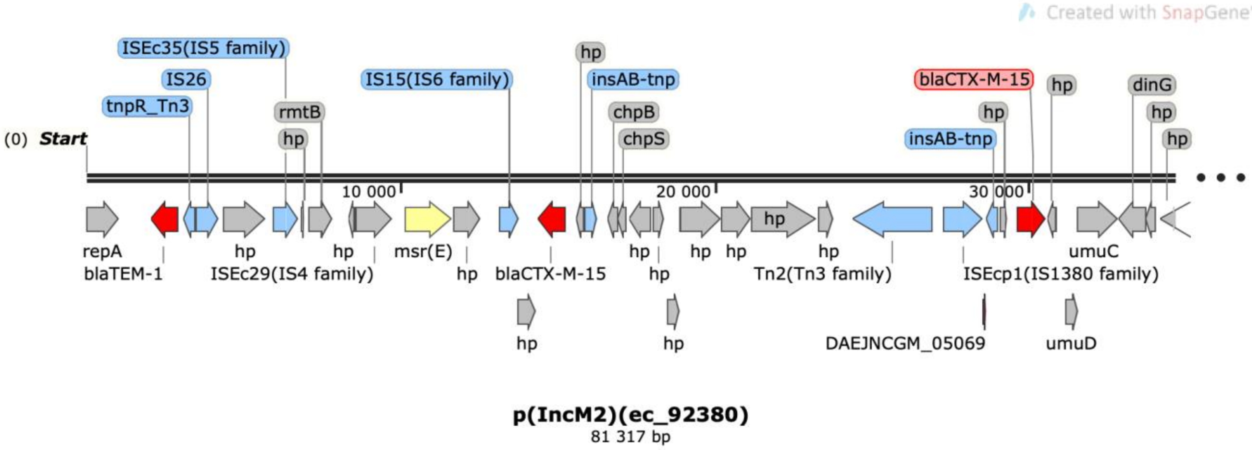

Figure S2. Structure of repeat region in IncM2 plasmid of *E. coli* ec_92380.

Open reading frames (ORFs) and their transcriptional orientation are shown by arrows with respective annotations. ORFs are colored as follows:, *bla*$_{TEM-1}$ and *bla*$_{CTX-M-15}$ (red), other ARGs (light yellow) , IS/Tn elements (light blue), other genes (grey). Delta (Δ) indicates partial deletion.
